# Tongxinluo Exerts Inhibitory Effects on Pyroptosis and Amyloid-*β* Peptide Accumulation after Cerebral Ischemia/Reperfusion in Rats

**DOI:** 10.1155/2021/5788602

**Published:** 2021-09-16

**Authors:** Bing Wang, Zhongkuan Lyu, Yuanjin Chan, Qiyue Li, Li Zhang, Kaili Liu, Yaming Li, Zhonghai Yu

**Affiliations:** ^1^Department of Traditional Chinese Medicine, Shanghai Jiao Tong University Affiliated Sixth People's Hospital, Shanghai 200233, China; ^2^Geriatrics Department of Chinese Medicine, Huadong Hospital, Fudan University, Shanghai 200040, China

## Abstract

Amyloid-*β* peptide (A*β*) accumulation is a detrimental factor in cerebral ischemia/reperfusion (I/R) injuries accounting for dementia induced by ischemic stroke. In addition to blood brain barrier (BBB), the glymphatic system mediated by aquaporin-4 (AQP-4) on astrocytic endfeet functions as an important pathway for the clearance of A*β* in the brain. Cerebral I/R induced astrocytic pyroptosis potentially causes the AQP-4 polarization loss and dysfunctional BBB-glymphatic system exacerbating the accumulation of A*β*. Furthermore, A*β* toxicity has been identified as a trigger of pyroptosis and BBB damage, suggesting an amplified effect of A*β* accumulation after cerebral I/R. Therefore, based on our previous work, this study was designed to explore the intervention effects of Tongxinluo (TXL) on astrocytic pyroptosis and A*β* accumulation after cerebral I/R in rats. The results showed that TXL intervention obviously alleviated the degree of pyroptosis by downregulating expression levels of cleaved caspase-11/1, N-terminal gasdermin D, nucleotide-binding oligomerization domain-like receptors pyrin domain containing 3 (NLRP3), interleukin-6 (IL-6), and cleaved IL-1*β* and abated astrocytic pyroptosis after cerebral I/R. Moreover, TXL intervention facilitated to restore AQP-4 polarization and accordingly relieve A*β* accumulation around astrocytes in ischemic cortex and hippocampus as well as the formation of toxic A*β* (A*β*_1–42_ oligomer). Our study indicated that TXL intervention could exert protective effects on ischemic brain tissues against pyroptotic cell death, inhibit astrocytic pyroptosis, and reduce toxic A*β* accumulation around astrocytes in cerebral I/R injuries. Furthermore, our study provides biological evidence for the potential possibility of preventing and treating poststroke dementia with TXL in clinical practice.

## 1. Introduction

Ischemic stroke, a common cerebrovascular disease, constitutes approximately 80% of stroke cases and is among the leading causes of long-term disability and dementia worldwide [1, 2]. For patients suffered from acute cerebral ischemia, the most important rescue measure is to restore blood flow of the ischemic cerebral tissue in a short-time window. However, the severe injuries following ischemia/reperfusion (I/R) greatly influence the therapeutic effects of reperfusion, and one of the major mechanisms lies in the neuroinflammation-related accumulations of toxic metabolites represented by amyloid-*β* peptide (A*β*) [3–5]. As a detrimental factor exacerbating cerebral I/R injuries, A*β* massively accumulates around astrocytes in ischemic brain tissues, accounting for the occurrence of dementia induced by ischemic stroke [4–7].

Recently, research studies have revealed that pyroptosis, a proinflammatory programmed cell death distinguished from apoptosis, plays a crucial role during I/R injuries and that gasdermin D (GSDMD) acts as its key effector by forming nanopores to damage cytomembrane integrity [8–10]. Canonical pyroptosis relies on the activation of nucleotide-binding oligomerization domain-like receptors pyrin domain containing 3 (NLRP3)/caspase-1 inflammasome, resulting in the cleavage of GSDMD and the secretion of proinflammatory cytokines such as interleukin-1*β* (IL-1*β*) [11]. However, in the caspase-4/5/11 (caspase-4/5 in humans, and the orthologous caspase-11 in rodents) mediated noncanonical pyroptosis pathway, GSDMD is the direct substrate of caspase-11, and the N-terminal fragment (GSDMD-N) from the full length GSDMD (GSDMD-FL) cleaved by caspase-11 is critical for the formation of nanopores leading to cell death, and meanwhile, as the upstream signaling molecule, GSDMD-N activates the NLRP3/caspase-1 inflammasome, causing the maturation and secretion of IL-1*β* [12].

Pyroptosis accounts for neuroinflammation after cerebral I/R in acute ischemic stroke [2, 10]. Thus, pyroptosis signaling molecules are becoming potential therapeutic targets to reduce cerebral I/R injuries and neuroinflammation-related accumulations of toxic metabolites including A*β*. Aquaporin-4 (AQP-4) is a water channel physiologically located with high polarization on the endfeet of astrocytes and functions as the main component of both the glymphatic system and blood brain barrier (BBB) which are major pathways for the clearance of A*β* in the brain [5]. Recently, our study revealed that astrocytic pyroptosis is a considerable trigger of AQP-4 polarization loss and BBB-glymphatic dysfunctions which promotes A*β* accumulation [13]. Furthermore, A*β* has been identified as a trigger of pyroptosis [14, 15], suggesting an amplified effect of A*β* accumulation after cerebral I/R.

According to the collateral disease theory of traditional Chinese medicine (TCM), the pathogenesis of acute ischemic stroke consists in stagnancy of collateral-Qi in a deficiency condition. Tongxinluo (TXL), formulated upon the collateral disease theory [16], possesses efficacies of supplementing Qi and promoting circulation of Qi and blood to dredge collaterals. TXL is approved by the State Food and Drug Administration of China in 1996 (state medical license no. Z20060322) and has become a common TCM prescription which is widely used for the prevention and treatment of cardiocerebrovascular diseases of blood stasis syndrome including ischemic stroke.

In modern research studies about TCM compound prescriptions for the prevention and treatment of cerebrovascular diseases, TXL is a typical representation showing beneficial effects such as BBB protection [17]. As is acknowledged, inflammation is closely related with blood stasis [18–20]. Our studies have indicated that TXL has multiple therapeutic effects against cerebral I/R injuries including antineuroinflammation [16, 21, 22]. Therefore, based on our previous work, the present study was designed to explore the potential intervention effects of TXL on pyroptosis and A*β* accumulation induced by cerebral I/R.

## 2. Materials and Methods

### 2.1. Drug and Preparation

TXL, dried superfine powder mixture of 12 components (Table 1), was provided by Shijiazhuang Yiling Pharmaceutical Incorporated Company (Shijiazhuang, Hebei, China). TXL was authenticated and standardized upon marker compounds in the Chinese Pharmacopoeia (2005, 2015). Moreover, ingredients of TXL were carefully analyzed and quality controlled by gas chromatography (GC) as well as high-performance liquid chromatography (HPLC) as described previously [23, 24]. TXL superfine power was dissolved in distilled water with the concentration of 0.1 g/ml (w/v), and then, the suspension was stored at 4°C until being used.

### 2.2. Animals

**Figure 1:**
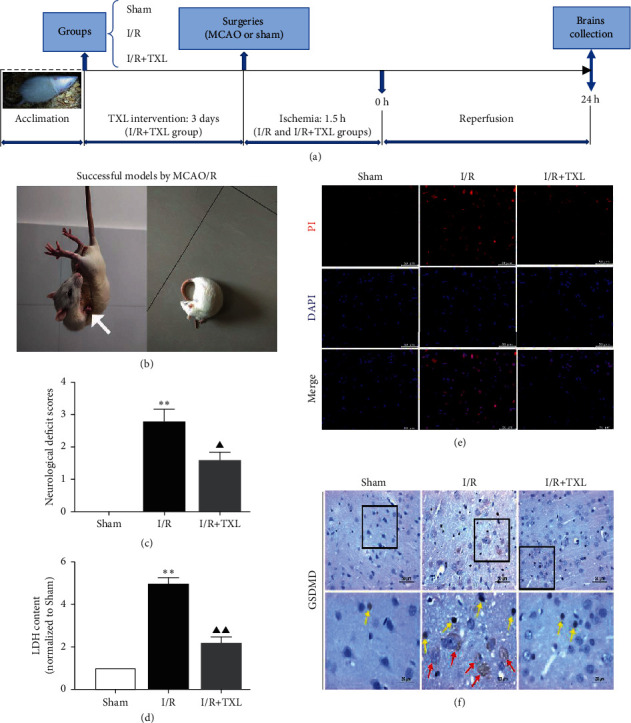
Schematic diagram of the experimental protocols (a) and assessments on neuroprotective and pyroptosis-inhibiting effects of TXL against cerebral I/R injuries (b–f). (b) Neurological deficits of successful MCAO/R rats with failure to extend right forepaw (white arrow) and circling to the right. (c) Neurological deficit scores of each group, *n* = 10. (d) Detection of LDH content, *n* = 4. (e) Representative pictures of PI immunofluorescent staining. The red dots represent the amount of nuclear PI uptake; scale bars, 50 *μ*m. (f) Representative pictures of GSDMD-FL (yellow arrows) and GSDMD-N (red arrows) immunohistochemistry staining; scale bars, 50/20 *μ*m. Data are presented as mean ± SEM. ^*∗∗*^*P* < 0.01 versus the sham group. ^▲^*P* < 0.05 and ^▲▲^*P* < 0.01 versus the I/R group.

A total of thirty-five male Sprague-Dawley rats, weighing 200–230 g, were obtained from Shanghai Laboratory Animal Research Center. They were housed in animal rooms of Shanghai Jiao Tong University Affiliated Sixth People's Hospital, under the standard laboratory conditions with controlled humidity and constant temperature. All rats were provided unlimited food and water with their acclimation for several days before experiments. Both animal handling procedures and experimental protocols (Figure 1(a)) were consistent with the guidelines for the management of laboratory animals and approved by the Animal Ethics Committee of Shanghai Jiao Tong University Affiliated Sixth People's Hospital.

### 2.3. Groups and Drug Administration

Our previous studies showed that the dosage of TXL administrated to rats at 1.6 g/kg/day was the optimal dosage for maximal protective effects on cerebral tissues suffered I/R injuries [16, 21]. Accordingly, we selected this optimal dosage of TXL for the current study, and the rats were randomly divided into the sham group (sham), cerebral ischemia/reperfusion group (I/R), and TXL administration group (I/R + TXL). The rats in the I/R + TXL group were administered orally with TXL suspension two times a day at 9 : 00 and 16 : 00 for three days before surgery and until they were sacrificed. For other two groups, rats were given the equivalent volume of distilled water.

### 2.4. Focal Cerebral I/R Models and Neurological Deficit Scores

The method of left middle cerebral artery occlusion/reperfusion (MCAO/R) was used for building focal cerebral I/R models of rats as described in our previous work [16]. Rats in I/R and I/R + TXL groups were subjected to MCAO/R (1.5 h ischemia and 24 h reperfusion) surgeries, while rats in the sham group only underwent the same operation with no insertion of the monofilament. Neurological examinations were performed after reperfusion as previously described [21]. Briefly, A 5-point scale was applied to assess the neurological deficits [25]: 0, no deficit; 1, failure to extend right forepaw; 2, circling to the right; 3, falling to the right; and 4, no spontaneous walking with a depressed level of consciousness. In the present study, rats at least with failure to extend right forepaw or circling to the right were considered as successful focal cerebral I/R models (Figure 1(b)), and five rats without any detectable neurological deficits after MCAO/R surgery were excluded from the following experiment. During the whole course, rectal temperature and cardiovascular rate of all rats were monitored and maintained.

### 2.5. Western Blotting Analysis

After 24 h reperfusion, the rats were deeply anesthetized, and their brains were quickly removed following cardiac perfusion with 200 ml normal saline. The levels of pyroptosis-related proteins and A*β*_1-42_ oligomers were detected by Western blotting. In brief, after concentrations measurement and protein denaturation, equal amounts of protein samples extracted from ischemic penumbra and equivalent area under sham were subjected to 10% sodium dodecyl sulfate-polyacrylamide gel electrophoresis (SDS-PAGE) and then transferred onto the polyvinylidnene fluoride membranes (Millipore, Billerica, MA, USA). Subsequently, the membranes were blocked at room temperature with 5% bovine serum albumin (BSA) for 1 h and incubated with the following primary antibodies at 4°C overnight: anti-GSDMD, anti-*β*-actin (CST, Danvers, MA, USA), anticaspase-11, anti-IL-6, anti-IL-1*β* (Santa Cruz, Dallas, TX, USA), anti-NLRP3, anticaspase-1 (Proteintech, Rosemont, IL, USA), and anti-A*β*_1–42_ (Abcam, Cambridge, UK). Then, the membranes were washed and incubated with corresponding secondary antibody (SAB, College Park, MD, USA) for 1 h at room temperature. After developing by the enhanced chemiluminescence kit (Millipore), pictures were captured with a gel imaging instrument (BioRad Laboratories, USA), and the intensities were analyzed by ImageJ software (National Institutes of Health, USA).

### 2.6. Lactate Dehydrogenase (LDH) Assay

Briefly, homogenates from cortex tissues in ischemic penumbra and equivalent area under sham were centrifuged, and then, the supernatant was used to detect the content of LDH for preliminarily evaluating the degree of pyroptosis by an LDH assay kit (Beyotime, Shanghai, China) following the manufacturer's instructions.

### 2.7. Immunofluorescence and Immunohistochemistry

After anesthetization followed by infusion with normal saline and then 4% paraformaldehyde, the brains were removed and immersed in 4% paraformaldehyde for 24 h fixation, and subsequently, paraffin slices (5/10 *μ*m) were prepared. Then, after dewaxing and rehydration, propidium iodide (PI) immunofluorescent staining of brain slices was performed. For the staining of objective proteins, the slices went through antigen retrieval, permeation by 0.3% triton-X 100, and then blockage with 5% BSA. Subsequently, the slices were incubated with the first antibodies for glial fibrillary acidic protein (GFAP) mixed, respectively, with GSDMD, NLRP3 (Proteintech), caspase-11, AQP-4, and A*β* (Santa Cruz) overnight at 4°C. After incubation with secondary antibodies and DAPI staining, the slices were covered with antiquenching agent for capturing fluorescent pictures by a laser scanning confocal microscope (Leica, Germany).

Immunohistochemical staining was used for observing the expression and location of GSDMD to assess pyroptosis. Briefly, the brain slices were dewaxed and rehydrated and went through antigen retrieval, permeation, inactivation of the endogenous catalase by H_2_O_2_, and then blockage with 5% BSA. Subsequently, the slices were incubated with anti-GSDMD for 2 h followed by secondary antibody for 1 h at room temperature. Then, 3,3-diaminobenzidine tetrahydrochloride and hematoxylin were used as color developing reagents for visualizing the slices. Pictures were captured with a light microscope (Leica, Germany).

### 2.8. Statistical Analysis

All the data were collected as the mean ± SEM. Statistical analysis was performed using GraphPad Prism 8.0 (GraphPad Software Inc., USA). Statistical significance of difference among groups was analyzed by one-way ANOVA or unpaired Student's *t*-test. *P* < 0.05 was considered to be statistically significant.

## 3. Results

### 3.1. TXL Inhibited the Cleavage of GSDMD and Alleviated the Degree of Pyroptosis after Reperfusion

Neurological functions and pyroptosis at 24 h after cerebral I/R were assessed. In accord with our previous studies, the present study showed that TXL exerted neuroprotective effects against cerebral I/R injuries (*P* < 0.05, Figure 1(c)). Compared with the I/R group, the I/R + TXL group showed lower amount of LDH content in the ischemic brain tissue (*P* < 0.01, Figure 1(d)) and decreased immunostainings of PI, GSDMD-FL and GSDMD-N (Figures 1(e) and 1(f)), indicating that TXL could inhibit the cleavage of GSDMD and thus alleviated the degree of pyroptosis after cerebral I/R.

### 3.2. TXL Inhibited Astrocytic Pyroptosis Mediated by the Activation of the Caspase-11/GSDMD Pathway

**Figure 2:**
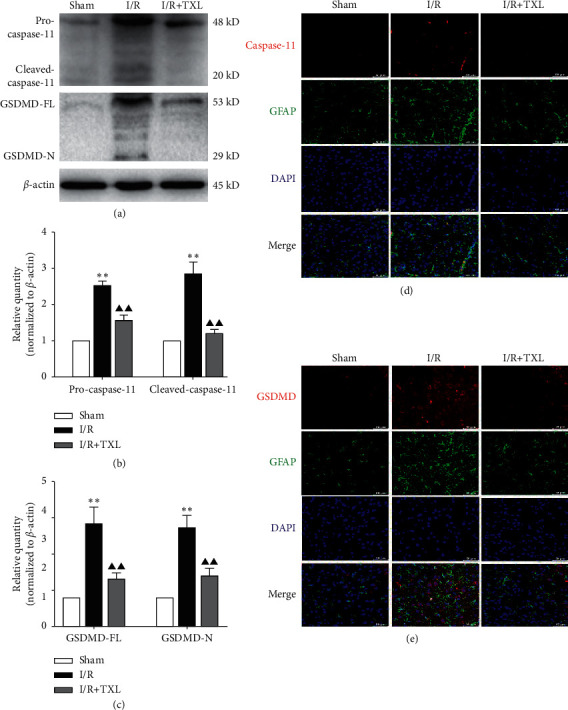
Caspase-11/GSDMD mediated pyroptosis in astrocytes and intervention effects of TXL. (a–c) Protein levels of pro/cleaved caspase-11 and GSDMD-FL/N in each group; *n* = 6. Data are presented as mean ± SEM. ^*∗∗*^*P* < 0.01 versus the sham group. ^▲▲^*P* < 0.01 versus the I/R group. (d-e) Representative pictures of double immunofluorescence staining of GFAP (green, the biomarker of astrocytes) with caspase-11/GSDMD (red), respectively; scale bars, 50 *μ*m.

Compared with the sham group, the I/R group showed higher protein levels of pro/cleaved caspase-11 and GSDMD-FL/N (*P* < 0.01 for all cases, Figures 2(a)–2(c)) and more immunofluorescent colocalizations of GFAP (biomarker of astrocytes), respectively, mixed with caspase-11 and GSDMD, which could be obviously reduced by TXL intervention (Figures 2(d) and 2(e)), indicating that TXL could exert inhibitory effects on astrocytic pyroptosis mediated by the activation of the caspase-11/GSDMD pathway.

### 3.3. TXL Alleviated Pyroptosis-Related Inflammatory Responses

**Figure 3:**
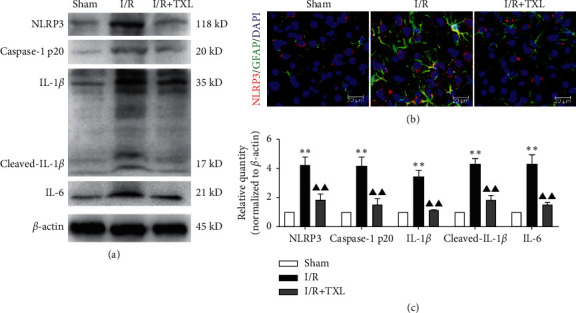
Intervention effects of TXL on pyroptosis-related inflammatory responses after reperfusion. (a, c) Protein expression levels of NLRP3, caspase-1 p20, IL-1*β*, and IL-6 in each group. Data are presented as mean ± SEM. ^*∗∗*^*P* < 0.01 versus the sham group. ^▲^*P* < 0.05 and ^▲▲^*P* < 0.01 versus the I/R group. (b) Representative pictures of the double immunofluorescence staining of GFAP (green) and NLRP3 (red); scale bars, 20 *μ*m.

Our study further explored the effects of TXL on pyroptosis-related neuroinflammation. The results showed that protein levels of NLRP3, caspase-1 p20, IL-1*β*, and IL-6 in the I/R group were significantly higher than those in the sham group (*P* < 0.01 for all cases), while TXL intervention could significantly downregulate expression levels of these pyroptosis-related inflammatory biomarkers (*P* < 0.01 for all cases) (Figures 3(a) and 3(c)). Moreover, Figure 3(b) exhibits the obvious double immunofluorescence colocalization of GFAP and NLRP3 after reperfusion, further indicating the activation of pyroptosis-related inflammatory responses in astrocytes of ischemic brain tissues, which could be inhibited by TXL intervention.

### 3.4. TXL Restored AQP-4 Polarization Loss and Reduced Toxic A*β* Accumulation after Reperfusion

**Figure 4:**
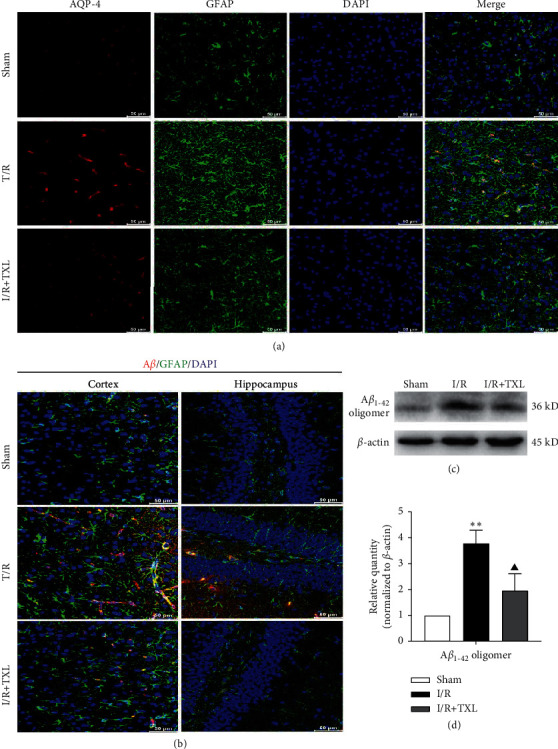
Intervention effects of TXL on AQP-4 polarization loss and A*β* accumulation after reperfusion. (a) Representative pictures of the double immunofluorescence staining of AQP-4 (red) and GFAP (green) in ischemic cortex; scale bars, 50 *μ*m. (b) Representative pictures of the double immunofluorescence staining of A*β* (red) and GFAP (green) in ischemic cortex and hippocampus; scale bars, 50 *μ*m. (c-d) Protein levels of A*β*_1–42_ oligomers in each group; *n* = 6. Data are presented as mean ± SEM. ^*∗∗*^*P* < 0.01 versus the sham group. ^▲^*P* < 0.05 versus the I/R group.

Furthermore, our results exhibited the loss of AQP-4 polarization with obvious dispersion in the ischemic cortex (Figure 4(a)) and the A*β* accumulation around astrocytes in ischemic cortex and hippocampus areas after reperfusion (Figure 4(b)), while TXL intervention could abate these pathological states (Figures 4(a) and 4(b)). In addition, the formation of A*β*_1–42_ oligomers (the main form of toxic A*β*) in the I/R group correspondingly increased compared with that in the sham group (*P* < 0.01), and TXL could markedly reduce the content of A*β*_1–42_ oligomers in ischemic brain tissues after reperfusion (*P* < 0.05) (Figures 4(c) and 4(d)).

## 4. Discussion

The accumulation of A*β* is the key pathological factor causing Alzheimer's disease (AD) which is the dominant type of dementia. Emerging research studies have indicated that neuroinflammation-related A*β* massively accumulates around astrocytes in ischemic brain tissue after cerebral I/R and accounts for the occurrence of dementia induced by ischemic stroke [6, 7, 26]. Pyroptosis is recognized as the important trigger of neuroinflammation during cerebral I/R injuries [10]. Consequently, pyroptosis is becoming a potential therapeutic target to treat neuroinflammation and A*β* accumulation after cerebral I/R.

According to TCM theories, the basic pathogenesis of dementia lies in deficiency of marrow sea and disuse of spirit caused by cerebral malnutrition with marrow sea shrivelled resulting from insufficiency of essence, Qi, and blood or by obstruction of Qi, fire, phlegm, and blood stasis in the brain. In addition to marrow sea deficiency, spleen-kidney deficiency, and turbid phlegm obstructing the brain, blood stasis is regarded as the main syndrome pattern of dementia [27]. Disturbance of microcirculation represented by BBB during cerebral I/R injuries is the pathological basis of ischemic stroke-induced dementia of blood stasis syndrome. Besides, research studies also revealed that thrombogenesis after cerebral I/R is the potential source of A*β* accumulation in brain tissues including capillaries [28].

TXL contains various natural medicines to exert efficacies of strongly dredging brain collaterals by invigorating Qi, removing blood stasis, expelling wind, as well as promoting the circulation of Qi by aromatic herbs. Accordingly, TXL is especially suitable for the prevention and treatment of cerebrovascular diseases including ischemic stroke and dementia with blood stasis in brain collaterals. Abundant evidences demonstrated that TXL has multiple therapeutic effects against cerebral I/R injuries [16, 17, 22]. Our previous work indicated that not only can TXL reduce death of brain cells but also alleviate the neuroinflammation caused by I/R injuries [21], implying that TXL may exert inhibitory effects on pyroptosis and A*β* accumulation after reperfusion which has not yet been demonstrated.

Pyroptosis is characterized by numerous nanopores on the cytomembrane formed by the cleaved GSDMD that leads to cellular swelling and death as well as releases of proinflammatory mediators [29]. LDH and PI staining detections are the effective methods used for assessing the degree of pyroptosis [30]. In our current study, the results showed that the amount of LDH content in ischemic tissues and PI staining after reperfusion obviously increased, while TXL intervention markedly lowered the increase, showing the potential inhibitory effects of TXL on cerebral I/R-induced pyroptosis. Currently, increasing research studies are targeting GSDMD as a strategy for the prevention and treatment of cerebral I/R injuries [10, 31]. We found that TXL intervention significantly reduced the translocation of cleaved GSDMD (GSDMD-N) into the plasma membrane area in ischemic brain tissues of rats, which further indicated the inhibitory effects of TXL on pyroptotic cell death induced by cerebral I/R.

Emerging studies have revealed that both noncanonical pyroptosis mediated by caspase-11/GSDMD and canonical pyroptosis by activation of NLRP3 are involved in I/R injuries [8, 32]. In this study, we observed the markedly upregulated expression levels of pro/cleaved caspase-11, GSDMD-FL/N, NLRP3, cleaved caspase-1, and proinflammatory mediators such as IL-1*β* and that GFAP (biomarker of astrocytes) obviously colocalized with caspase-11, GSDMD, and NLRP3, respectively. Furthermore, our results showed that TXL intervention could significantly inhibit astrocytic pyroptosis associated with inactivating the caspase-11/GSDMD pathway and alleviated pyroptosis-related inflammatory responses after reperfusion.

As the main component of both the glymphatic system and BBB, AQP-4 is a water channel physiologically located with high polarization on the astrocytic endfeet to facilitate A*β* clearance in the brain [33], and thus, astrocytic pyroptosis becomes the potential cause of AQP-4 polarization loss, BBB damage, and glymphatic dysfunction which promote A*β* accumulation around astrocytes. On the other hand, studies have indicated that A*β* toxicity acts as a trigger of pyroptosis and BBB damage [14, 15, 34]. Therefore, a vicious circle is considered to form between astrocytic pyroptosis and A*β* accumulation in cerebral I/R injuries, exacerbating BBB damage which is a vital trigger of both AD and vascular dementia (VD), the two main types of dementia [35]. In the present study, our results exhibited the AQP-4 polarization loss with obvious dispersion accompanied with A*β* accumulation around astrocytes in ischemic cortex and hippocampus as well as increased A*β*_1–42_ oligomers (the main form of A*β* toxicity) after cerebral I/R. While TXL intervention could obviously restore AQP-4 polarization and abate A*β* accumulation and the formation of A*β*_1–42_ oligomers, indicating the blocking effects of TXL on the potential vicious circle between the astrocytic pyroptosis and A*β* accumulation after reperfusion, which provides not only detailed action principle for protective effects of TXL against cerebral I/R induced BBB damage but biological evidence for the potential efficacy of preventing and treating poststroke dementia by TXL in clinical practice.

In summary, based on the previous work, our present study further demonstrated that TXL could protect ischemic brain tissues against pyroptotic cell death and pyroptosis-related neuroinflammation, inhibit astrocytic pyroptosis by inactivating caspase-11/GSDMD, and reduced toxic A*β* accumulation around astrocytes in cerebral I/R injuries, and thus potentially contribute to the prevention and treatment of poststroke dementia.

## Figures and Tables

**Table 1:** Composition of TXL.

Ingredients (Latin name)	Family	Chinese name	Part used	Voucher specimen number	Ratio (%)
Plants					
*Dalbergia odorifera* T. Chen	Leguminosae	Jiang Xiang	Heartwood of stem and root	11,005	4.000
*Boswellia carteri* Birdw	Burseraceae	Ru Xiang	Resin	11,006	5.927
*Borneolum syntheticum*	Dipterocarpaceae	Bing Pian	Resin	11,007	3.626
*Panax ginseng* C.A. Mey	Araliaceae	Ren Shen	Root and rhizome	11,001	1.667
*Paeonia lactiflora* Pall.	Ranunculaceae	Chi Sao	Root	11,003	1.558
*Ziziphus jujuba* Mill. Var. spinosa (Bunge) Hu H.F. Chou	Rhamnaceae	Suan Zao Ren	Seed	11,002	1.173
*Santalum album* L.	Santalaceae	Tan Xiang	Heartwood of stem	11,004	0.354

Insects					
*Cryptotympana pustulata* Fabricius	Cicadidae	Can Tui	Skin	12,005	18.111
*Hirudo nipponica* Whitman	Hirudinidae	Shui Zhi	Dried body	12,004	27.330
*Steleophaga plancyi* (Boleny)	Corydiidae	Tu Bie Chong	Female dried body	12,003	18.111
*Buthus martensii* Karsch	Buthidae	Quan Xie	Dried body	12,002	18.111
*Scolopendra subspinipes mutilans* L. Koch	Psittacidae	Wu Gong	Dried body	12,001	3.623

## Data Availability

The data used to support the findings of this study are available from the first authors upon request.

## Abbreviations

A*β*: Amyloid-*β* peptide
AD: Alzheimer's disease
AQP-4: Aquaporin-4
BBB: Blood brain barrier
GFAP: Glial fibrillary acidic protein
GSDMD: Gasdermin D
IL-1*β*: Interleukin-1*β*
IL-6: Interleukin-6
I/R: Ischemia/reperfusion
LDH: Lactate dehydrogenase
MCAO/R: Middle cerebral artery occlusion/reperfusion
NLRP3: Nucleotide-binding oligomerization domain-like receptors pyrin domain containing 3
PI: Propidium iodide
TCM: Traditional Chinese medicine
TXL: Tongxinluo.
